# Review: Epigenetic mechanisms in ocular disease

**Published:** 2013-03-21

**Authors:** Shikun He, Xiaohua Li, Nymph Chan, David R. Hinton

**Affiliations:** 1 Department of Pathology, Keck School of Medicine of the University of Southern CA; 2 Department of Ophthalmology, Keck School of Medicine of the University of Southern CA; 3 Doheny Eye Institute, Los Angeles, CA; 4 Henan Eye Institute, Zhengzhou, China

## Abstract

Epigenetics has become an increasingly important area of biomedical research. Increasing evidence shows that epigenetic alterations influence common pathologic responses including inflammation, ischemia, neoplasia, aging, and neurodegeneration. Importantly, epigenetic mechanisms may have a pathogenic role in many complex eye diseases such as corneal dystrophy, cataract, glaucoma, diabetic retinopathy, ocular neoplasia, uveitis, and age-related macular degeneration. The emerging emphasis on epigenetic mechanisms in studies of eye disease may provide new insights into the pathogenesis of complex eye diseases and aid in the development of novel treatments for these diseases.

## Introduction

Epigenetic mechanisms influence gene expression and function without modification of the base sequence of DNA and may be reversible, heritable, and influenced by the environment [1,2]. They include DNA methylation, post-translational histone modifications, chromatin remodeling, and deployment of non-coding RNA [1-3]. Epigenetic mechanisms play a role in the pathogenesis of major human diseases [4] such as cardiovascular disease [5], diabetes [6], neurodegenerative disease [7], and cancer [8,9]. Breakthroughs in epigenetics will help us understand complex biologic phenomena associated with development [10,11], inflammation [12-14], aging [15], stem cell biology [16], immunity [17], and angiogenesis [18]. This review provides evidence that the pathogenesis of complex eye diseases such as corneal dystrophy, glaucoma, uveitis, cataract, diabetic retinopathy, and age-related macular degeneration (AMD) is regulated by epigenetic mechanisms. Ultimately, these basic studies will be translated into novel therapies; epigenetic drugs are currently in clinical trials, most notably in treating cancer [19,20].

### Factors mediating epigenetic regulation

The chromatin structure provides the context for gene expression: transcriptional activity diminishes with increased chromatin density, while enhanced transcriptional activities are associated with a loosening of chromatin structure [1]. Such changes in the state of chromatin are affected by DNA methylation, histone modification, and non-coding RNA.

DNA methylation is catalyzed by DNA methyltransferases (DNMTs), which have an additional methyl group at the 5-position of cytosine that converts the cytosine to 5-methylcytosine (5-mc) [1]. 5-mc is known as the fifth base of the genome. More recently, 5- (hydroxymethyl) cytosine (5-hmc), the sixth base of the genome [21,22], and 5-formylcytosine and 5-carboxylcytosine, the seventh and eighth bases, were discovered [23,24]. The CpG dinucleotide is the most important site of DNA methylation. In general, CpG methylation silences genes while demethylation activates them; however, recent studies have shown that the functional effects of DNA methylation can vary according to the genomic context [25].

Histone is subjected to various post-translational modifications, including acetylation, methylation, phosphorylation, ubiquitination, and sumoylation. These modifications occur primarily within the N-terminal tails of histones protruding from the surface of the nucleosome, as well as on its core region [26]. These modifications and recognition modules lead to the establishment of histone code and create an epigenetic mechanism for regulating various physiologic and pathological phenomena. In general, histone acetylation activates gene expression, and histone deacetylation suppresses gene expression [27].

Non-coding RNA includes short interference RNA (siRNA), microRNA (miRNA), Piwi-interacting RNA (piRNA), and long non-coding RNA [1,3,28]. miRNA regulates gene expression in various ways, such as directly binding to DNA or binding to the gene promoter region; the influences of miRNA on gene expression are diverse and complex [29]. Importantly, circulating miRNAs have been identified as biomarkers for human diseases [30].

### International epigenetic research organizations and programs

The Association for the Study of the Epigenome in Europe was established in 1999, and launched the Human Epigenome Project (HEP) in 2003. The National Human Genome Research Institute (NHGRI) launched a public research consortium named ENCODE, the Encyclopedia Of DNA Elements in September 2003 to carry out a project to identify all functional elements in the human genome sequence. An association concerned with epigenetic research in Asia was jointly established by China, South Korea, Japan, and Singapore in 2006. The National Eye Institute, since its inception, has engaged in a planning effort to present current advances in ocular studies, and to identify and prioritize the goals in vision research (NEI planning). In the institute’s latest issue of “Vision Research: Needs, Gaps and Opportunities,” completed in August 2012, epigenetic mechanisms were discussed regarding disorders of the retina, cornea, and lens (Vision Research 2012). Furthermore, the world's largest epigenetic research project was initiated recently. The study will include 5,000 pairs of twins, who will be studied to investigate how different phenotypes occur in identical twins [31]. In 2010, the International Human Epigenome Consortium (IHEC) was launched to coordinate international collaborative efforts to produce reference maps of epigenomes for cellular states relevant to human health and disease. With recent reports from the ENCODE project consortium showing that 80% of the genome is functional, the significance of research into epigenetic mechanisms has become even more important [32].

## Discussion

### Epigenetics and keratitis

The interaction between pathogens (e.g., bacteria, viruses, and fungi) and immune cells results in the activation of inflammatory gene expression. Although there is little information on the participation of epigenetic factors in bacterial keratitis, the possible role of epigenetic mechanisms in the pathogenesis of bacterial infections in other systems has been investigated. Lipopolysaccharide (LPS), which is part of the structure of certain bacteria, was found to increase histone deacetylase (HDAC) activity. Inhibition of HDAC decreases LPS-stimulated tumor necrosis factor (TNF) expression caused by the accumulation of nuclear factor kappa B (NF-κB)/p65 at the TNF promoter. Interestingly, HDAC3 regulates TNF production in cardiomyocytes [33]. Bacteria can induce inflammatory signaling through the interaction of microbial associated molecular patterns with Toll-like receptors and subsequently activate the mitogen-activated protein (MAP) kinases (MAPK) cascade and NF-κB, leading to increased production of inflammatory cytokines such as interleukin (IL)-12, IL-6, and TNF. More importantly, the production of inflammatory cytokines is under the control of histone acetylation/deacetylation [34].

Herpetic keratitis is a common infectious corneal disease. Herpes simplex virus 1 (HSV1) can infect corneal epithelial cells and sensory neurons to establish a latent infection, leading to recurrence of HSV1 in the cornea when the virus is activated by various stimulatory factors. Gene replication is activated during acute infection; but instead of HSV1 viral DNA being transcribed into RNA and viral RNA being translated into viral proteins, only the latency-associated transcription factor is persistently expressed, and thus, latency is maintained. Therefore, understanding the mechanism by which HSV1 is maintained in latent infection and how HSV1 is activated is critical for controlling HSV infection [35,36].

Recent research indicates that the establishment of latency and the reactivation of HSV1 are tightly regulated by epigenetic mechanisms [35,36]. In acute infection, the replication of HSV1 requires participation of the transcription factor HIRA, histone H3 acetylation, and H3K4 methylation. During latent infection, H3K9 and K27 methylation is the major event of histone modifications; in the transition from latency to active infection, H3K9/14ac and H3K4me are the dominant histone modifications. The latency-associated transcript increases deposition of heterochromatic H3K9me2, H3K9me3, and H3K27me3 and reduces the formation of H3K4me3 on lytic gene promoters, which indicates that histone methylation is important in maintaining HSV1 latency [35,36].

At present, there is no effective therapy for latent infection. However, recent research shows that latent HSV1 infection can be activated by the application of the HDAC inhibitor trichostatin A (TSA) [37]; the reactivated virus could then be killed using specific anti-HSV treatment, suggesting that epigenetic therapy is a promising new approach in the treatment of latent HSV infection.

There are no reports on the role of epigenetic factors such as DNA methylation and histone acetylation in the pathogenesis of fungal keratitis; however, fungal metabolic products may interact with Toll-like receptors (TLRs), causing a decrease in histone acetylation and an increase in HDAC expression, in a manner similar to that of bacterial infection [34], and then activate downstream NF-κB signaling, leading to the production of inflammatory factors that promote the development of fungal keratitis. We further speculate that the level of histone acetylation in fungal keratitis is low; therefore, fungal keratitis could be inhibited by increasing histone acetylation with histone deacetylation inhibitor. This implies that the disequilibrium between histone acetylation and deacetylation may be a potentially important mechanism in the pathogenesis of keratitis.

### Epigenetics in amblyopia and myopia

Histone acetylation/deacetylation may play an important role in the pathogenesis of form deprivation amblyopia [38]. A rat model of form deprivation amblyopia was produced by eyelid suture. Daily intraperitoneal administration of the histone deacetylase inhibitors valproic acid or sodium butyrate resulted in recovery of visual acuity and tested visual evoked potentials (VEPs) almost to the same level as the controls after the sutures were removed. The result demonstrates that epigenetic factors are involved in the development of experimental form deprivation amblyopia, and suggests that inhibition of histone deacetylation might help to prevent visual loss in this disorder [38].

In a recent article, Zhou et al. reported that the expression of collagen 1α1 (*COL1A1*) mRNA was reduced during induction of form deprivation myopia in mice, whereas the frequency of methylation in CpG islands of the collagen 1 promoter was increased compared with control eyes [39]. Importantly, during recovery, the expression of *COL1A1* mRNA was increased, corresponding to a decrease in CpG methylation. The results indicate that higher levels of DNA methylation in the *COL1A1* promoter may inhibit scleral collagen synthesis and contribute to the development of myopia [39].

### Epigenetics and cataract

Multiple factors play important roles in cataract formation, including genetic, metabolic, nutritional, and environmental factors; cataract may also develop secondary to other systemic diseases or syndromes [40]. Epigenetic factors may also be involved in cataract formation [41]. Brg1 is a tumor suppressor that is part of the SWF/SNF family. This complex has ATPase activity and regulates chromatin remodeling, thus playing a role in inhibiting or activating the transcription of multiple genes. Using dominant negative Brg1 transgenic mice with a lens-specific promotor, He et al. showed that the transgenic mice developed cataract, while the lenses in the control group were transparent. The mechanism of the changes was thought to be related to the role of Brg1 in lens fiber differentiation and denucleation [41].

DNA methylation, and one of the DNA-methylation-associating proteins, methylation binding protein 2 (MeCP2), may play an important role in transforming growth factor (TGF)-β-induced posterior capsular opacification (PCO) after cataract surgery. Importantly, the use of the DNA methylation inhibitor zebularine can inhibit lens epithelial-myofibroblastic transformation in vitro [42]. This result suggests that aberrant DNA methylation may be relevant to PCO; additionally, methylation inhibitors may potentially be used to treat PCO [42].

### Epigenetics and glaucoma

Multiple factors play important roles in the development of glaucoma and retinal ganglion cell death. These factors include predisposing single nucleotide polymorphisms (SNPs) and environmental effects [43]. A better understanding of the mechanisms involved in the onset and progression of glaucoma is crucial to the development of better therapies. Recent evidence shows that HDAC 2 and 3 transcripts are significantly increased after acute optic nerve injury (ONI); in contrast, histone H4 acetylation in retinal ganglion cells was decreased following ONI, suggesting a correlation between increased HDAC activities and ONI [44]. In addition, Fem1cR is expressed in the early stage of neuronal cell apoptosis; the death of retinal ganglion cells is closely related to the silenced Fem1cR gene and increased HDAC3 activity in mice [45].

Additional experiments show that the application of histone deacetylase inhibitors such as TSA and valproic acid can reduce the loss of ganglion cells or can even enhance axonal regeneration after optic nerve damage [46]. These reports suggest that abnormal histone acetylation/deacetylation may be related to retinal ganglion cell damage in glaucoma. Furthermore, significant differences in genomic DNA methylation have been found in peripheral mononuclear cells from patients with open angle glaucoma compared with healthy controls [47]. In the future, genome-wide mapping of the changes in DNA methylation, histone modifications, and the expression of miRNA in human retinal ganglion cells will help us to determine the profile of epigenetic aberrations in glaucoma.

### Epigenetics and proliferative vitreoretinopathy

The epithelial-mesenchymal transition of retinal pigment epithelial (RPE) cells into myofibroblast-like cells plays a key role in the pathogenesis of proliferative vitreoretinopathy (PVR). TGF-β is a major inducer of this process, and α-smooth muscle actin (SMA)-positive RPE cells have been shown to promote PVR membrane contraction that leads to retinal detachment [48]. In addition, studies in other cell types and disorders have shown that wound healing is regulated by epigenetic factors, including DNA methylation and histone acetylation [49-51]. Of particular note, MeCP2 is a key regulator of epithelial-myofibroblast transformation [49]. Recent reports indicate that the balance between histone acetylation and deacetylation is lost in many fibrotic disorders [50]. HDAC inhibitors suppress renal fibrosis induced by diabetes or TGF-β [50]. The HDAC inhibitor TSA also reduces platelet-derived growth factor–induced fibroblast proliferation [51].

### Epigenetics and retinitis pigmentosa

Retinitis pigmentosa (RP) is a heritable, degenerative retinal disease that causes progressive visual impairment and blindness. Many RP gene mutations have been identified, but the mechanism leading to photoreceptor death is still unclear, and no treatment is available for most patients [52]. A recent study found that an increase in HDAC activity is observed before photoreceptor degeneration in the rd1 mouse model of RP, and that the degeneration can be reduced by applying HDAC inhibitors through upregulating peroxisome proliferator-activated receptor γ [53]. miRNA have also been implicated in photoreceptor degeneration. Notably, if the retinal DICER enzyme is specifically knocked down in mice, a reduced electroretinography response is observed in degenerated retinal cells [54]. In addition, reduced expression of miR-96, miR-182, and miR-183 is found in rd1 mice compared with normal mouse retinas, and the expression of miR-96, miR-183, miR-1, and miR-133 [55] is aberrant in transgenic mice with the Pro347Ser mutation in rhodopsin compared with wild-type mice.

Recently, valproic acid, an HDAC inhibitor, has been used for treating patients with retinitis pigmentosa [56]. Although encouraging preliminary results were shown, the benefit of this drug in RP needs to be confirmed in a placebo-controlled clinical trial.

### Epigenetics and diabetic retinopathy

Poor glycemic control (PC) is associated with many complications, including diabetic retinopathy (DR). Recently, a role for epigenetics in the pathogenesis of diabetic complications has been proposed [57]. In human umbilical vein endothelial cells (HUVECs), a heightened glucose level increases the expression and binding of the histone acetyltransferase p300 to the promoters of endothelin-1, fibronectin, and vascular endothelial growth factor (VEGF) [58]. In streptozotocin (STZ)-treated rats, the retinas and retinal endothelial cells (RECs) from animals kept in PC show increased expression of HDAC1, HDAC2, and HDAC8, and a reduction in the activity of a histone H3-specific acetyltransferase; these changes were not reversed when the PC rats were returned to good glycemic control. The result suggests that the epigenetic metabolic memory phenomenon may be the major reason for the continuation of DR even when the blood glucose level returns to normal [59].

Alterations in miRNA expression have also been observed in diabetic eyes. When rats treated with STZ were compared to untreated rats, changes in expression were detected in 37 miRNAs. Six of the miRNAs with confirmed alterations were differentially expressed over the course of STZ-induced diabetes [60]. In another study, VEGF-induced miR-17–5p, miR-18a, miR-20a, miR-21, miR-31, and miR-133 expression was observed in the RECs of STZ-treated rats. The p53-responsive miR-34c was also detected, implicating miRNAs in mediating the proangiogenic or proapoptotic effects caused by VEGF and p53 [61]. Reduced miR-200b and increased VEGF have been observed in HUVECs and bovine RECs treated with high glucose. Further, knocking down miR-200b inhibits the diabetes-induced upregulation of p300 in the retina, implying crosstalk between two epigenetic mechanisms in diabetic retinopathy [62].

### Epigenetics and age-related macular degeneration

Age-related macular degeneration (AMD) manifests as choroidal neovascularization (CNV) in the wet form and geographic atrophy (GA) in the late dry form [63]. Recently, epigenetic mechanisms have been implicated in the pathogenesis of AMD [64,65]. Hypoxia-inducible factor-1α (HIF-1α) has been suggested to contribute to the pathogenesis of AMD [66]. Epigenetic regulation of HIF-1α has been evaluated in cell culture and cancer models. The expression of HIF-1α can be reduced via HDAC1 by upregulating p53 and the Von Hippel–Lindau protein, through which the expression of VEGF is also inhibited [67]. HDAC7 associates with HIF-1α to increase HIF-1α’s transactivation ability [68], but VEGF induces the nuclear exit of HDAC7 to activate proangiogenic gene expression [69]. The HIF-1-directed hypoxic response can be regulated by histone methylation as well [70]. In a retinal ischemic rat model, TSA not only protected the retina from ischemic damage but also inhibited the TNF-α induction of matrix metalloproteinase-1 and matrix metalloproteinase-3 [71]. The pathogenesis of AMD may also potentially be regulated by DNA methylation. Clusterin/apolipoprotein J may have either anti- or proangiogenic activities and has been found in drusen [72-74]. Clusterin contains CpG islands in its promoter region, and treatment of ARPE-19 cells with the DNA methylation inhibitor 5-azacytidine (5-AZA) with or without HDACi upregulated clusterin expression [74]. In a study mapping promoter DNA methylation in AMD and age-matched normal RPE/choroid samples, the antioxidants glutathione S-transferase isoforms mu1 and mu2 were downregulated and heavily methylated in their promoter regions in AMD samples. Additionally, the proangiogenic angiopoietin-like protein 2 had less methylation in its promoter in the AMD samples [75]. Hypomethylation of the interleukin-17 receptor C (*IL17RC*) promoter has recently been identified in peripheral blood cells from patients with AMD and was associated with increased expression of IL17RC in their peripheral blood and affected retina and choroid. These results suggest that epigenetic regulation of *IL17RC* may play a role in the pathogenesis of AMD [76].

Regulation of gene expression by miRNA is also involved in CNV. In a laser-induced murine CNV model, the intravitreal injection of pre-miR-21 significantly diminished CNV volume [77]. When mice were put under ischemic stress, the injection of pre-miR-31 or −150 caused significant downregulation of VEGF in the retina, while premiR-31 also reduced the expression of retinal HIF-1α and platelet-derived growth factor B. The injection of all three of the same pre-miRs, or of pre-miR-31 or −150 by itself reduced CNV lesion sizes in a laser-induced CNV mouse model, while the levels of these three miRNAs were significantly reduced in CNV lesions [78]. Other mechanisms involving miRNAs could play a part in either form of AMD. When miR-23a is downregulated, the death receptor Fas is upregulated, resulting in RPE cell apoptosis [79]. The expression of miR-155 is induced by TNF-α, IL-1β, and interferon-γ at moderate levels, individually or synergistically, in combination via the Janus kinase/signal transducers and activators of transcription pathway [80]. In the aged retina, upregulated expression of miRNA-9, miRNA-125b, miRNA-146a, and miRNA-155 has been found, all of which were responsive to the NF-κB activation that modulates amyloidogenesis by inhibiting TSPAN12 and that modulates innate immunity by downregulating complement factor H [81]. DICER1, which is part of the miRNA-processing machinery, is downregulated in human GA eyes. In animal and cell culture experiments, the depletion of DICER1 reduces RPE cell viability by causing the accumulation of *Alu* RNA, which is toxic to RPE cells. A mouse model with a DICER knockdown in the retina displayed an RPE degeneration phenotype similar to human GA [82].

### Epigenetics and retinoblastoma

Retinoblastoma (RB) is the most common intraocular tumor in children. Recent studies indicate that in addition to RB1 gene mutation, tumor development also involves promoter DNA methylation of other tumor suppressor genes. Whole-genome sequencing analysis from samples of patients with RB and normal controls showed that the tumors contained a small number of mutations or chromosomal rearrangements; more likely, RB1 mutation causes epigenetic abnormalities in cancer-related genes, namely, the high expression of spleen tyrosine kinase (SYK), suggesting the regulation of RB and SYK is closely related [83]. In addition, an association between RB and hypermethylation of the RAS association domain family 1A gene (*RASSF1A*) promoter has been demonstrated [84]. Taken together, these findings indicate that epigenetic mechanisms participate in the pathogenesis of RB.   

### Epigenetic and uveal melanoma

Previous studies showed that methylation of RASSF1A promoter CpG island is a common event in uveal melanoma; and importantly, hypermethylation of RASSF1A is related to the development of metastatic disease [85-88]. However, a recent study demonstrated that the human telomerase reverse transcriptase gene was methylated, but not on RASSF1A, in uveal melanoma [89]. The discrepancy may be due to genetic heterogeneity in human uveal melanoma. More research is needed to identify these different patterns of DNA methylation.

In addition to DNA methylation, histone acetylation has also been implicated in the pathogenesis of uveal melanoma. In vitro, histone deacetylase inhibitors can inhibit the metastatic activity of uveal melanoma cell by inhibiting cell proliferation and inducing apoptosis, which is similar to the Fas-dependent apoptosis pathway [90,91]. Additionally, it has been suggested that HDAC inhibitors reduce the invasiveness of uveal melanoma by inducing changes in DNA conformation, resulting in inhibited expression of some key tumor genes, reduced invasiveness of the tumor cells, and blockage of tumor cell proliferation [91].

### Epigenetics and ocular stem cells

The potential for self-renewal and differentiation in stem cells, including embryonic stem cells and induced pluripotent stem cells, has become an active area of epigenetics research [16]. The dynamic regulation of stem cells by epigenetic factors may play an important role in stem cell renewal and differentiation [92]. Genes associated with self-renewal are silenced in the process of stem cell differentiation, while genes that regulate cell differentiation are activated; these stem cell functional phenomena are regulated by epigenetic factors. It has been suggested that reprogramming of promoter methylation is one of the key determinants of the epigenetic regulation of pluripotency genes [93]. Shen et al. found that approximately 1.4% of CpG islands have undergone significant re-methylation in the differentiation of embryonic stem cells into neural stem cells [94]. In the murine retina, increased methylation corresponds to lower levels of EphA5 receptor mRNA expression in Müller glial stem cells in the mouse retina [95]. The expression of Sirt1 (one of the HDACs) mRNA in retinal stem cells was significantly decreased with increasing age [96]. In addition, miRNA maintains stem cells in an undifferentiated state [97]. Adult stem cells originating from the eye, including corneal epithelial and endothelial stem cells, trabecular meshwork stem cells, and retinal stem cells, in theory, may have characteristics similar to those of other stem cells in the human body, where their mechanisms for differentiation and self-renewal are regulated by epigenetic factors.

### Epigenetics and pharmacotherapeutics

Pharmaceutical agents may also be viewed as environmental factors with widespread impact on the human body. For example, many drug-metabolizing enzymes, gene therapy vectors, and drug targets are subjected to regulation by epigenetic factors [98]. The resistance of viruses or bacteria to antiviral and antibiotic drugs may be related to aberrant epigenetic regulation, which is relevant in clinical practice [99]. Another common phenomenon is individual differences in reactions to drugs. DNA methylation plays an important role, especially in regulating certain drug metabolizing enzymes in the cytochrome superfamily [100]. The differences in expression of cytochrome c P450 are responsible for different responses to the same drug in different individuals, and P450 expression is regulated by DNA methylation [100]. Attention should be paid to these epigenetic factors in the development of ocular therapeutics and the personalized treatment of ocular diseases.

### Future perspectives 

The rapid increase in epigenetic research in the past decade has increased our understanding of the role of epigenetic mechanisms in human physiology and disease [4]. Improvements in technology have resulted in the ability to perform individual-based human DNA methylation mapping (human DNA methylome) [101]. With the expansion of epigenetic research, several new concepts and terms have emerged, such as the epigenome, epigenetic epidemiology, epigenetic pathology, epigenetic disease, epimutation, and epigenomic therapy [102]. Although considerable progress has taken place, challenges and questions remain. What are the epigenetic maps of the various types of ocular cells and how do they vary among individuals and in disease? Which epigenetic factors in complex eye diseases play a key role, and which play a secondary role? What are the epigenetic marks that predict progression in blinding eye disease? How do epigenetic factors regulate ocular stem cells and tissue regeneration? More specifically, what are the roles of histone modifications and non-CpG methylation and 5-(hydroxymethyl) cytosine methylation in eye development and disease?

Ultimately, the goal of such research is to find effective therapies for blinding eye disease. Although epigenetic therapeutic agents such as 5-AZA and suberoylanilide hydroxamic acid are currently being investigated in human clinical trials for cancer [19,20], a major problem in applying epigenetic agents for ocular disease is the lack of target cell or target gene specificity. Consideration should be given to the development of small molecules that specifically target epigenetic alterations related to specific eye diseases.
